# A New Concept of Health Can Improve the Definition of Frailty

**DOI:** 10.1007/s00223-015-0038-x

**Published:** 2015-07-28

**Authors:** Maarten Boers, Alfonso J. Cruz Jentoft

**Affiliations:** VU University Medical Center, Amsterdam, The Netherlands; Hospital Universitario Ramón y Cajal, Madrid, Spain

**Keywords:** Health, Frailty, Sarcopenia, Opsteoporosis, Gerontolog

## Abstract

Following a newly developed concept of health, this viewpoint suggests that the concept of frailty can usefully be defined as: the weakening of health, i.e. the resilience or capacity to cope, and to maintain and restore one’s integrity, equilibrium, and sense of wellbeing in three domains: physical, mental, and social.

## Introduction

 Frailty is a rapidly evolving concept in the field of Geriatric Medicine and Gerontology. The number of MedLine citations with the term “frailty” has grown exponentially since its first appearance in 1986. Frailty is strongly linked to the concepts of osteoporosis and sarcopenia. When first introduced, frailty described older people with multimorbidity who were disabled: restricted in their activities of daily living and in need of help from others, often living in institutions [1]. However, such a description was soon deemed too far ‘downstream’ to be useful in the quest to tackle disability early or to even prevent it in high risk individuals, a key objective in the Gerontological Sciences. Subsequently frailty was suggested to be a precursor state to disability [2]. The dynamic balance between different frailty states (being well/robust, frail but not dependent, or frail and dependent) was soon acknowledged [1]. The aim of this paper is to refine the definition of frailty by linking it to a new definition of health.

## Present Concepts of Frailty

Scientific progress in frailty is seriously hampered by the lack of consensus on its definition, in turn delaying development of screening and diagnostic tools, as well as treatment [3]. Definitions of frailty can broadly be grouped into two different conceptual models or frameworks.

The first framework, best described by the work of Rockwood et al. [4], states that diseases and impairments accumulate in an individual, and this accumulation predisposes to adverse outcomes, mainly death and hospitalization. This model is based on the idea of the collection of risk factors. A risk factor predisposes towards a health condition or outcome (death, hospitalization) in people not suffering from that condition. We use the term ‘health condition,’ defined as a situation of impaired health, because it is broader and thus more appropriate than the term ‘disease.’ Modifiable risk factors can be treated so that the condition or outcome is retarded in time or prevented (or the chance of its occurrence reduced).

The second model, based on the work by Fried et al. [5], uses a physiological background (the cycle of frailty) to describe a phenotype that may be identified in clinical practice. This approach considers frailty as a (medical or geriatric) syndrome. A syndrome is a set of symptoms and signs occurring together, constituting a health condition. People with a medical syndrome, typically called patients, suffer its impact; this ‘burden of disease’ can be identified and measured. Treatment of a syndrome is aimed at cure or improvement to remove or alleviate that burden and avoid progression of the condition. Often a currently present health condition can also be seen as a risk factor for future health conditions (for example, heart failure predisposes to community-acquired pneumonia [6]), but treatment is mostly focused on the current burden.

Thus, these models represent two different points of view on where to put the focus in the process leading from frailty to disability. The former model considers frailty solely as a ‘pre-clinical’ entity, i.e., a risk; the latter regards it primarily as a health condition, i.e., a progressive process to be staged across a range of severity states, including different degrees of disability. Somewhat confusingly in this second model frailty is also regarded as a risk for further disability and for other health conditions and outcomes [7, 8].

Currently, the predominant view on frailty is focused on the risk for future adverse outcomes, although this risk may be described by deficit accumulation or with a phenotype. This is the person who is still coping, but whose balance can be upset by a relatively small adverse occurrence to result in failure, often at several levels. Accordingly, the most widely used definition of frailty considers it “a biologic syndrome of decreased reserve and resistance to stressors, resulting from cumulative declines across multiple physiologic systems, and causing vulnerability to adverse outcomes” [5]. However, this definition appears conceptually suboptimal. For starters, the word ‘syndrome’ should be reserved for a phenotype that describes a set of symptoms and signs, constituting a health condition, as noted above. Second, it contains the clauses “decreased resistance to stressors” and “vulnerability to adverse outcomes” but the latter is no more than an incomplete operationalization of the former: when a patient is ‘vulnerable,’ a ‘stressor’ could induce a higher or more severe occurrence of a new health condition or worsening of an existing (chronic) condition. All of these equate to an adverse health outcome. Given the above, we felt the definition of frailty might benefit from a conceptual recalibration by applying a newly emerged concept of health.

## Linking the Frailty Concept to a New Definition of Health

The classic WHO definition of health as complete wellbeing has recently been challenged. Experts in this topic are proposing a change, with a new emphasis on the ability to adapt and self-manage in the face of social, physical, and emotional challenges [9]. This is a dynamic concept of health [10], linked to resilience and ability to cope.

If this definition of health is accepted, frailty could simply be defined by adding ‘the weakening of’ to it:

*Health is the resilience or capacity to cope, and to maintain and restore one’s integrity, equilibrium, and sense of wellbeing in three domains: physical, mental, and social.*

*Frailty is the weakening of (health; health is defined as) the resilience or capacity to cope, and to maintain and restore one’s integrity, equilibrium, and sense of wellbeing in three domains: physical, mental, and social.*

**Fig. 1:**
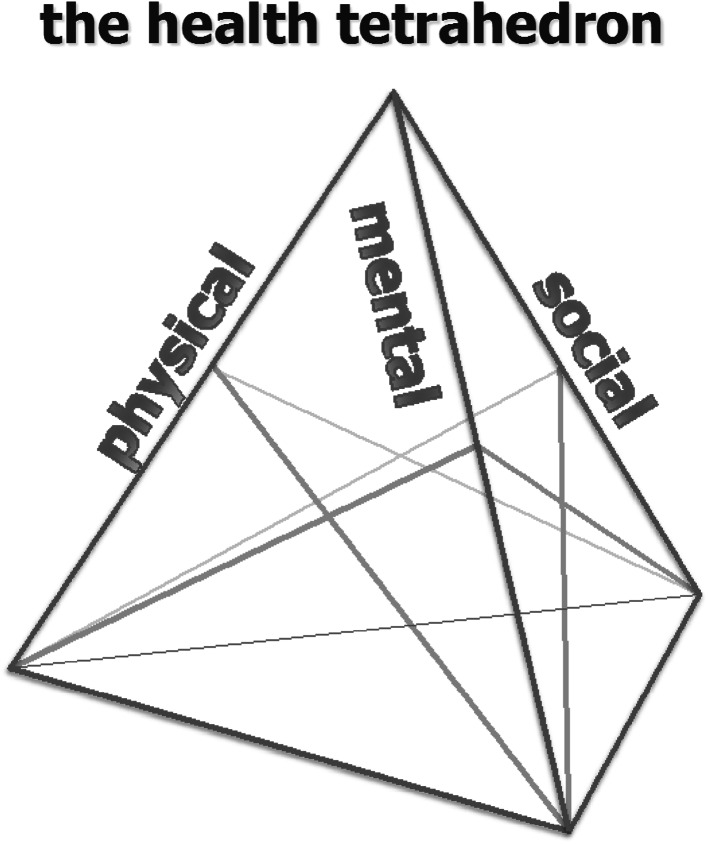
New concept of health depicted as tetrahedron

In this model, health can be viewed as a tetrahedron (Fig. 1) with three ribs and secondary supporting structures. One or more of the ribs can become weakened, and this can be partially compensated by the secondary supporting structures. The tetrahedron still stands (i.e., the patient is still coping and maintaining integrity) but is weakened, less able to withstand threats to the equilibrium (stressors). On further weakening of one or more of the ribs, when the supporting structures are also weak or nonexistent, the structure can be stressed beyond a point where it collapses (adverse health outcome), reversibly or irreversibly.

We suggest this definition of frailty linked to a general model of health creates a clear conceptual structure, but to be successful it also needs to be operationalized, a topic for future research. As with the concept ‘increased vulnerability’ in the previous definition, the term ‘weakening of the resilience’ needs to find the right comparison. This weakening, the reduced ability to cope, is an age-related phenomenon that links frailty and aging. So, should someone’s resilience be compared against the resilience of a young individual, or against some theoretical optimum defined for each age group? Or against resilience currently observed in a given population? Such questions are related to the more broad question and research agenda on what characterizes normal aging. Each approach to defining ‘normal’ resilience can be tested to see which works best in clinically relevant situations.

In summary, we propose that a conceptual definition of frailty based on the definition of health may be more applicable than concepts of frailty as a risk factor or a syndrome.
